# Measuring What Matters: Little Evidence Supporting the Content Validity of EQ-5D in People with Duchenne Muscular Dystrophy and Their Caregivers

**DOI:** 10.1177/0272989X211062237

**Published:** 2021-11-26

**Authors:** Philip A. Powell, Jill Carlton, Donna Rowen, John Brazier, Karen Facey, Klair Bayley, Fleur Chandler, Josie Godfrey, Emily Crossley

**Affiliations:** School of Health and Related Research, University of Sheffield, Sheffield, UK; School of Health and Related Research, University of Sheffield, Sheffield, UK; School of Health and Related Research, University of Sheffield, Sheffield, UK; School of Health and Related Research, University of Sheffield, Sheffield, UK; Usher Institute, University of Edinburgh, Edinburgh, UK; Duchenne Australia, Perth, Western Australia, Australia; Duchenne UK, London, UK; JG Zebra Consulting, London, Greater London, UK; Duchenne UK, London, UK

The recent article by Crossnohere et al. assessed the “appropriateness” of the EQ-5D for use as a measure of health status in Duchenne muscular dystrophy (DMD). This was investigated in terms of the instrument’s responsiveness (to differences in health status), convergent validity (correlation with disease-specific measures), feasibility and burden (how easy was the EQ-5D to understand and answer), and some minimal tests of content validity (did the participants think that the EQ-5D was consistent with “health status”). In their abstract, the researchers conclude that they “found support for the appropriateness of EQ-5D to assess health status in Duchenne.”^1^

We welcome the research by Crossnohere et al., but we would like to make explicit the caveat to their conclusion that the researchers conducted a very limited assessment of the content validity of the EQ-5D for use in measuring health status (or health-related quality of life, as used elsewhere in the article) in DMD. While this is acknowledged in the Discussion section of the article, it is not clear in the Methods section or in the abstract, and there is the concern that this caveat may therefore be lost on a more casual reader.

Content validity is regarded as the most important psychometric property of any patient-reported outcome measure (PROM) according to the widely respected COSMIN guidelines, which should necessarily extend to preference-based measures used to generate utilities (as a special category of PROMs).^2^ Put simply, before a measure is used to inform quality-adjusted life-years in cost-utility analysis, you would want to make sure you are measuring the right thing(s) (and in this context, when considering health-related quality of life, we argue that should be the domains that matter most to patients).

A fuller assessment of content validity would involve asking participants, usually in a more in-depth interview setting, whether the instrument is *comprehensive* (i.e., nothing important is missing), each item is *relevant* (i.e., applicable to the target population and context of use), and each item is *comprehensible* (i.e., understood as the developers or researchers intended). Crossnohere et al. rightfully acknowledge that “it is important to understand whether this generic measure [EQ-5D] is comprehensive, relevant and understandable to people with rare conditions.” However, the questions they used did not fully reflect this goal. First, no questions were asked about whether the EQ-5D was comprehensive. Second, participants were asked whether the EQ-5D was “consistent with health state of the person with Duchenne” (a majority agreed that it was) and “did or did not describe real health status” (of which 43% agreed). These questions do not ask about the relevance of each item, do not ask people to consider health-related quality of life, and may otherwise be difficult for lay people to understand (what is “health status”?). Finally, the authors do ask if the EQ-5D was “easy to understand” (but not whether each item was understood as intended).

Crossnohere et al. conclude their article recommending that “advocacy groups look holistically at addressing the barriers to access of therapies in rare diseases such as Duchenne, rather than honing in specifically on perceived shortcoming of the EQ-5D.” We would extend this to say that all stakeholders need to consider how value is determined in access decisions and that, for rare diseases, where there is a paucity of clinical evidence and knowledge, modeling of value must capture elements that are most important to patients, including effects on quality of life.^3^ The Duchenne UK Project HERCULES initiative has worked holistically over the past three years with all stakeholders to develop better understanding of the burden of illness with DMD and the sufficiency of current quality-of-life measures.^4^ This multifaceted work has shown potential cause for concern over the use of the EQ-5D in DMD. A recent systematic review showed unsatisfactory comprehensiveness of the EQ-5D in DMD based on the available evidence, which is notably limited.^5^ Moreover, qualitative work from the project demonstrated that certain domains of the EQ-5D may not be relevant for all people with DMD, such as the mobility domain focusing wholly on walking (and not using mobility aids, such as wheelchairs).^6^ As a consequence, a condition-specific PROM and preference-based measure has been developed based on in-depth qualitative interviews with people with DMD, designed to have greater content validity: the DMD-QoL and DMD-QoL-8D.^7,8^

In summary, while we welcome Crossnohere et al.’s contribution, we would like to emphasize to readers that no conclusions can yet be drawn that the EQ-5D is *measuring what matters* to people with DMD and their caregivers with regard to health-related quality of life (or the “quality” in a quality-adjusted life-year). We argue that content validity should be a fundamental aspect in determining the appropriateness of any outcome measure. Therefore, we recommend that evidence on the content validity of the instrument is considered alongside evidence of other psychometric properties to make conclusions on the appropriateness of EQ-5D for use in DMD.
